# Estimating Magnetic Field at Joint Centers Reduces Kinematic Errors in Inertial Motion Capture

**DOI:** 10.21203/rs.3.rs-9337794/v1

**Published:** 2026-04-08

**Authors:** Six Skov, Keenon Werling, Johanna O’Day, Jennifer Hicks, C. Karen Liu, Scott Delp

**Affiliations:** Department of Mechanical Engineering, Stanford University, Stanford, CA 94305 USA; Department of Computer Science, Stanford University, Stanford, CA 94305 USA; Department of Bioengineering, Stanford University, Stanford, CA 94305 USA; Department of Bioengineering, Stanford University, Stanford, CA 94305 USA; Department of Computer Science, Stanford University, Stanford, CA 94305 USA; Department of Bioengineering, Stanford University, Stanford, CA 94305 USA; Department of Mechanical Engineering, Stanford University

**Keywords:** Gait analysis, Inertial measurement unit, Joint angles, Kalman filter, Kinematics

## Abstract

Inertial measurement units (IMUs) are widely used to measure human motion, but accuracy remains inferior to gold-standard optical motion capture. Traditionally, researchers estimate joint angles from IMUs using global sensor fusion methods that assume the measured acceleration is gravity and the measured magnetic field is magnetic north. However, these assumptions are frequently violated, when linear accelerations are significant and magnetic fields are distorted. Recent magnetometer-free methods improve accuracy by replacing the gravity assumption with a more dynamically consistent assumption of a shared acceleration at the joint center, but these methods are prone to drift error. To improve accuracy, we developed the Magnetic Field at Inertial Joint Center (MAJIC) filter, which leverages both the common acceleration and magnetic field at a joint center. The magnetic field is adaptively included when necessary to reduce drift. We evaluated the MAJIC filter’s estimated lower extremity joint kinematics against optical motion capture for 11 participants performing 20 minutes of ambulation tasks. The MAJIC filter produced joint angles with a median root mean squared error (RMSE) of 7.1°, compared to global sensor fusion methods (9.3°) and recent magnetometer-free methods (7.5°). The MAJIC filter also had a smaller range of RMSEs over all joints (5.5° to 11.4°) compared to global fusion methods (6.4° to 22.8°) and magnetometer-free methods (4.9° to 20.5°). We hope these improvements, and the open-source implementation of this filter, advance the measurement of human motion outside of the laboratory.

## I. Introduction

LONG-duration, out-of-lab motion capture is critical for bringing biomechanical insights into real-world applications such as fall prevention, personalized training, and assistive device control. At present, IMUs are the most practical and commercially available solution for real-time motion sensing in real world settings. Cameras offer one alternative, but restrict capture volume and introduce privacy issues [1], [2], [3]. New sensing modalities, such as Wi-Fi-based motion reconstruction and wearable flexible sensors, remain in early development [4], [5]. While IMUs show promise for a variety of applications [6], [7], [8], [9], [10], [11], [12], [13], the accuracy of these systems is lower than optical motion capture, with errors of 5° to 10° per joint axis [14], [15]. Reducing the accuracy gap between in-lab and out-of-lab motion capture would increase confidence for real-world applications, while also unlocking wider scale data collection for machine learning and other data driven methods.

The traditional method for estimating a joint angle with IMUs involves estimating the global orientation of each segment and subsequently calculating the relative orientations between them [16], [14], [17], [18]. Sensor fusion algorithms estimate global orientation by integrating gyroscope readings over time, then applying a correction based on a measured acceleration (assumed to be gravity) and magnetic field reading (assumed to be magnetic north) to mitigate drift [19], [20], [21], [22]. The accuracy of methods based on global orientation suffers both in and out of laboratory settings due to violations of these two key assumptions.

First, magnetometers measure the local magnetic field, which is easily distorted by ferrous materials or electronics, invalidating the ”magnetic north” assumption [23], [24], [25], [26]. These distortions are frequently non-linear, further violating the assumption that the field is constant over time and uniform in space. To address the violation of the magnetic field being constant over time, some methods adaptively omit the magnetometer based on variance over time [22], [27]. However, these filters do not address the variance of magnetic distortion in space and struggle to reconcile the magnetic fields at different body segments into a single global magnetic north, two common challenges when using IMUs for biomechanics applications.

Second, human motion often involves significant linear accelerations, invalidating the ”gravity-only” accelerometer assumption. Methods developed for joints with a single degree of freedom [28] and with three degrees of freedom [29] replace the assumption of a shared ”gravity” with the assumption of a shared acceleration at the joint center, estimated from each segment using rigid body dynamics. This class of filters directly computes relative orientations, without estimating the global orientation or using global references. These latter methods exclusively use gyroscopes and accelerometers, re moving the magnetometer altogether. Without an additional reference, like magnetic north, the relative orientation is ambiguous about the axis defined by the acceleration, similar to the ambiguous heading associated with using only gravity to define a global orientation. Due to this ambiguity, magnetometer-free methods can suffer from drift. For motions where the acceleration changes direction, the drift error is drastically reduced, as observations of acceleration at the joint center over time can be considered distinct sensors to disambiguate the rotation. However, when the accelerations at the joint center are small—such as when the body segment is not moving—or always in the same direction, drift cannot be mitigated [30].

We built on the work in [29] to develop and share the Magnetic Field and Acceleration at Inertial Joint Center (MAJIC) filter that leverages magnetic field and acceleration at the joint center to directly estimate the relative orientation of two segments. In this way, the MAJIC filter aims to address two types of error: global methods’ error due to magnetic distortion in space and magnetometer-free methods’ error due to drift. Rather than assuming the magnetometer represents a uniform magnetic north, we only assume that it represents the magnetic field at the joint center, and that the field gradient across the joint is negligible. This localized assumption allows us to exploit the magnetometer for drift correction without needing to reconcile distortions at the whole body scale. Furthermore, MAJIC adaptively includes this magnetic field data only when the relative orientation is not fully determined by acceleration alone. We evaluated our proposed algorithm, along with a traditional global filter and a magnetometer-free relative filter, on the dataset from [14], which includes optical motion capture and IMU readings for the trunk and lower extremities on 11 participants who each performed 20 minutes of ambulation tasks. To support reproducibility and widespread access, we provide an open-source implementation of all presented methods along with the dataset used for evaluation.

## II. Methods

### A. The MAJIC Filter

We developed the **MAJIC filter**, an error-state, relative orientation Kalman filter that directly estimates the relative orientation R*^JK^* , for segments *J* and *K* connected by a ball joint, from the shared acceleration $\hat{\text{a}}$ and magnetic field $\hat{\text{b}}$, where the hat ( ∧ ) designates the virtual sensor reading estimated at the joint center.

**Fig. 1.**
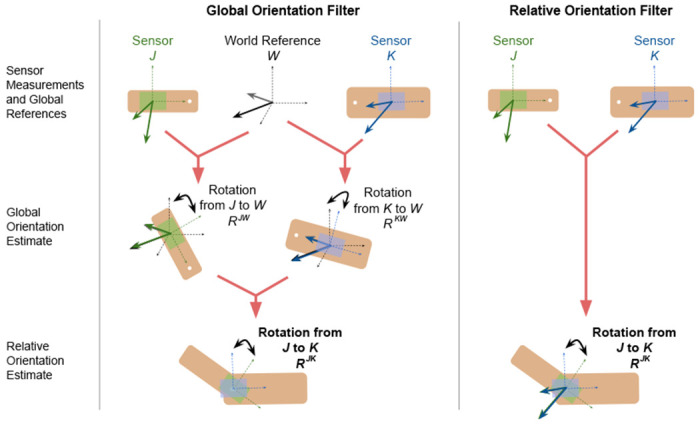
Procedure for estimating the relative orientation of two segments, R^JK^ using traditional global (left) and relative (right) Kalman filters. Global reference W, sensor J, and sensor K make measurements for the specified sensors. Then, sensor measurements are aligned in the specified filter to generate a global or relative orientation estimate. Relative orientation R^JK^ is estimated from the global orientation estimates, if necessary.

To construct a relative orientation using the MAJIC filter, we use a set of *N* sensors on segments *J* and *K* to collect measurements $y_{1\ldots N}^{J}$ and $y_{1\ldots N}^{K}$. Frequently, the set of *N* sensors consists of accelerometers and magnetometers, but this notation allows for other sensor modalities as well. We use the set of sensor readings to estimate virtual sensor readings at the joint center, ${\overset{ˆ}{y}}_{1\ldots N}^{J}$ and ${\overset{ˆ}{y}}_{1\ldots N}^{K}$ . These virtual sensor readings at the joint center, which we assume to be equal but observed in different sensor frames, are aligned using sensor fusion to directly estimate the relative orientation R*^JK^* (Figure 1 (right)). This differs from the traditional global orientation filter, which aligns sensor readings to global references, usually gravity and magnetic north, to create global orientations R*^WJ^* and R*^WK^* (Figure 1) (left); the global orientations are then combined to form the relative orientation R*^JK^* . The traditional approach carries error from the global orientation estimate into the final relative orientation, since the sensors measure linear acceleration instead of only gravity and a potentially distorted local field instead of the magnetic north. By avoiding the global references, relative filters avoid this error.

More specifically, the MAJIC filter estimates R*^JK^*, the relative orientation of two segments, in two steps: a prediction step using gyroscopes and an update step using acceleration and magnetic field at the joint center. During the prediction step, the segment orientations ${\tilde{R}}^{WJ}$ and ${\tilde{R}}^{WK}$ are estimated from previous orientation estimates and the measured angular velocity from the gyroscopes [29]. Then, during the update step, we estimate the error in the predicted orientations, denoted $\eta^{J}$ and $\eta^{K}$, from ${\overset{ˆ}{y}}_{1\ldots N}^{J}$ and ${\overset{ˆ}{y}}_{1\ldots N}^{K}$, measurements of the same values at the joint center in different reference frames. Given predicted orientations ${\tilde{R}}^{WJ}$ and ${\tilde{R}}^{WK}$, nominal errors $\eta^{J}$ and $\eta^{K}$ and sensor readings ${\overset{ˆ}{y}}_{1\ldots N}^{J}$ and ${\overset{ˆ}{y}}_{1\ldots N}^{K}$, we form the following expression

(1) $${\tilde{R}}^{WJ}\left(I_{3}+\left[\eta^{J}\times \right]\right){\overset{ˆ}{y}}_{1\ldots N}^{J}-{\tilde{R}}^{WK}\left(I_{3}+\left[\eta^{K}\times \right]\right){\overset{ˆ}{y}}_{1\ldots N}^{K}\approx 0$$

We use this expression to define the implicit measurement model for state x and measurements z as

(2) $$h(x,z)=h\left(\left[\eta^{J},\eta^{K}\right],\left[{\overset{ˆ}{y}}_{1\ldots N}^{J},{\overset{ˆ}{y}}_{1\ldots N}^{K}\right]\right)$$

(3) $$={\tilde{R}}^{WJ}\left(I_{3}+\left[\eta^{J}\times \right]\right){\hat{y}}_{1\ldots N}^{J}-{\tilde{R}}^{WK}\left(I_{3}+\left[\eta^{K}\times \right]\right){\hat{y}}_{1\ldots N}^{K}$$

After the prediction step, $\eta^{J}$ and $\eta^{K}$ reset to zero, and the measurement model becomes

(4) $$\tilde{h}=h\left([0,0],\left[{\overset{ˆ}{y}}_{1\ldots N}^{J},{\overset{ˆ}{y}}_{1\ldots N}^{K}\right]\right)={\tilde{R}}^{WJ}{\overset{ˆ}{y}}_{1\ldots N}^{J}-{\tilde{R}}^{WK}{\overset{ˆ}{y}}_{1\ldots N}^{K}$$

To estimate the update for $\eta^{J}$ and $\eta^{K}$ from the error in the measurement model $\tilde{h}$, we form the Jacobian of the measurement model with respect to the state $\left[\eta^{J},\eta^{K}\right]$,

(5) $$H=\frac{\partial h}{\partial \left[\eta^{J},\eta^{K}\right]}=\left[\begin{array}{cc} {\tilde{R}}^{WJ}\left[{\overset{ˆ}{y}}_{1}^{J}\times \right] & -{\tilde{R}}^{WK}\left[{\overset{ˆ}{y}}_{1}^{K}\times \right]\\ \ldots & \ldots \\ {\tilde{R}}^{WJ}\left[{\overset{ˆ}{y}}_{N}^{J}\times \right] & -{\tilde{R}}^{WK}\left[{\overset{ˆ}{y}}_{N}^{K}\times \right] \end{array}\right]$$

Using the evaluated measurement error $\tilde{h}$ from equation 4 and the measurement update Jacobian **H** from equation 5, we then estimate the near-optimal error state to be

(6) $$\left[\eta^{J},\eta^{K}\right]=K*\tilde{h}\left([0,0],\left[{\overset{ˆ}{y}}_{1\ldots N}^{J},{\overset{ˆ}{y}}_{1\ldots N}^{K}\right]\right)$$

where **K** is the Kalman gain defined by

(7) $$K=PH^{T}{\left(HPH^{T}+R\right)}^{-1}$$

with covariance of the state **P**, covariance of the sensor readings **R**, and the measurement update Jacobian **H**.

In the literature, acceleration is the only sensor reading that has been used to perform the update step in this form of Kalman filter, applying rigid body dynamics to estimate the acceleration at the joint center â from a measured acceleration a at a known offset [31]. However, when only using one sensor for $\overset{ˆ}{y}$, **H** is not a full rank matrix, and the update step is under-determined. While time continuity can constrain some of the ambiguity in the estimate, there are certain motions during which the acceleration information is insufficient to observe or unambiguously define the 3D relative orientation. The observability (*o^J^*) of a relative orientation, or the extent to which the acceleration measurement is sufficient to fully describe the relative orientation of two bodies, is defined

(8) oJ=aˆJ×dJaˆJdt+ωJ×aˆJ2

where ${\overset{ˆ}{a}}^{J}$ is the acceleration at the joint center estimated from sensors on segment $J,\omega_{J}$ is the measured angular velocity, and dJaˆJdt is the derivative of the acceleration at the joint center, all taken and expressed in the local frame *J* [30]. For a given joint, each segment produces an independent estimate of observability. Thus, we define the overall observability as the minimum of all the segment observability estimates.

When the observability of the joint drops below a threshold, we use both the acceleration at the joint center â and an estimate of the magnetic field at the joint center $\overset{ˆ}{\text{b}}$ as our set of sensors to make **H** full rank and fully constrain the relative orientation. The threshold used in our implementation is set to $150\frac{m^{2}}{s^{5}}$, the approximate lower bound for observability at the knee during motion in data from [30]. In this implementation, we assume the magnetic field is constant along the joint center projection, such that $b=\overset{ˆ}{b}$ for any given magnetometer. This is more dynamic than the assumption that the magnetic field is constant for all sensors, as discrepancies are resolved between adjacent segments rather than between all sensors on the body.

### B. Evaluation

We evaluated the MAJIC filter using paired optical motion capture and inertial measurement unit data published in [14]. This dataset includes IMU and optical markers on the torso, pelvis, thighs, shanks, and feet of 11 participants performing 10 minutes of walking and 10 minutes of other ambulation tasks. We computed segment-to-segment 3D relative orientations for the lumbar joint, hips, knees and ankles as angle axis rotations from between two segments. We constructed the offset from each sensor to the joint center using optical motion capture. We constructed the relative rotations from marker data and from IMU data with the following sensor fusion filters:

- **Extended Kalman Filter (EKF):** a traditional Kalman filter for the global orientation of each segment independently. Relative orientations are then constructed by multiplying the global orientations of the corresponding segments (see Figure 1, left).
- **Magnetometer-Off Relative Kalman Filter (Mag Off):** this filter is an implementation of the one presented in [29].
- **Magnetometer-On Relative Kalman Filter (Mag On):** the proposed filter, with the naive inclusion of the magnetometer.
- **Magnetometer Adaptive Relative Kalman Filter (MAJIC):** the proposed MAJIC filter, with an adaptive magnetometer based on the observability of the joint orientation.

Additional comparisons to other standard filters can be found in the Supplementary Materials [18], [21], [20]. The dataset and code implementation for this project can be found at https://github.com/osskov/MAJIC_mocap/.

We report the root mean squared error (RMSE) and Pearson correlation for each IMU method compared to the matched optical motion capture reference, pooled for each data capture (participant, joint, and trial). Pearson’s correlations are measured between the marker-based data and each IMU filter for each column of the relative orientation, expressed in angle-axis representation. Root mean squared error is similarly measured as the magnitude of the rotation difference between the marker-based orientation and the IMU-filter-based orientation, expressed in angle-axis representation. These errors represent a total 3D joint angle error, rather than separate sagittal, frontal and transverse angle errors, to comprehensively describe the total relative orientation error. To compare performance across the different estimation methods, we conducted a non-parametric repeated-measures analysis. We first used the Friedman test to determine overall significant differences between methods for RMSE, with each unique combination of participant, trial, and joint axis treated as a block. If a significant overall effect was detected (p < 0.05), we performed pairwise post hoc comparisons using the Wilcoxon signed rank test. To control for the family-wise error rate associated with multiple comparisons, all p-values from these pairwise tests were adjusted using the Holm-Bonferroni correction. A significance level of α = 0.05 was used for all analyses.

## III. Results

The MAJIC filter had the smallest median RMSE over all movement trials (7.07°), outperforming the Extended Kalman Filter (9.28°, *p* = 5.22*e*^−12^), Magnetometer On (7.35°, *p* = 1.44*e*^−9^), and Magnetometer Off (7.48, *p* = 9.02*e*^−2^) algorithms. All methods produced median RMSEs under 10°.

**Fig. 3.**
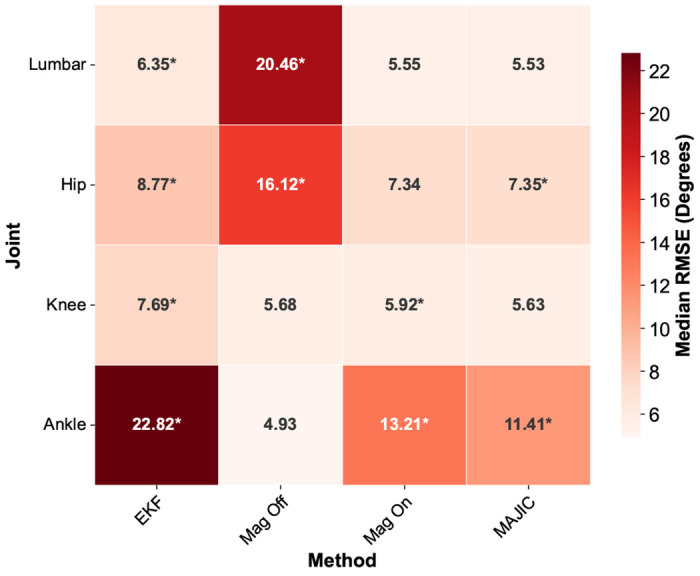
Median 3D joint angle RMSE per joint for each method. Methods which are significantly different from the best performing method at each joint are marked with an asterisk. * Significantly different from best method in row (p < 0.05, Wilcoxon)

The MAJIC filter had the smallest range in median RMSE across all joints (5.53° to 11.41°) compared to EKF (6.35° to 22.82°), Magnetometer Off (4.93° to 20.46°) and Magnetometer On (5.55° to 13.21°) (Figure 3). In addition to difference in range of RMSE across joints, the distribution of RMSE across joints differed by method. The Magnetometer Off had the largest median RMSE at the lumbar joint and the smallest at the ankle joint. The EKF, Magnetometer On, and MAJIC filter had largest error at the ankle joint, and smallest error at the lumbar joint. The MAJIC filter had a median RMSE that was as small or smaller than EKF and Magnetometer Off for all joints except the ankle (Figure 3). At the ankle, the MAJIC filter had a smaller median error than both EKF and Magnetometer On filters, but larger than the Magnetometer Off filter.

Correlation between joint angles estimated from optical motion capture and joint angles estimated from IMUs was generally high across joints, with the hip joint in particular showing strong agreement for all four methods (all above 0.93, Table I). The MAJIC filter achieved the highest mean correlation for the hip (0.96) and was tied for the highest mean correlation at the lumbar joint (0.89). Conversely, the Magnetometer Off filter demonstrated the strongest correlations for the distal joints, with higher correlations than all other methods at the knee (0.97) and ankle (0.96). The ankle joint proved to be the most challenging joint, with the EKF showing the poorest correlation by a large margin (0.62).

**Fig. 4.**
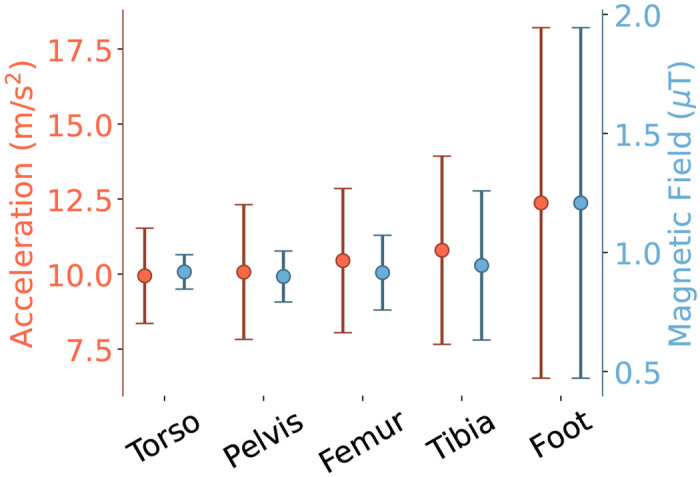
Mean and standard deviation of magnetometer and accelerometer reading magnitudes for each segment expressed in the global frame. Ideally, magnetic north and gravity would be vectors with a constant magnitude with no standard deviation at all segments. Both mean and standard deviation for all sensors increases in the more distal segments.

**Fig. 5.**
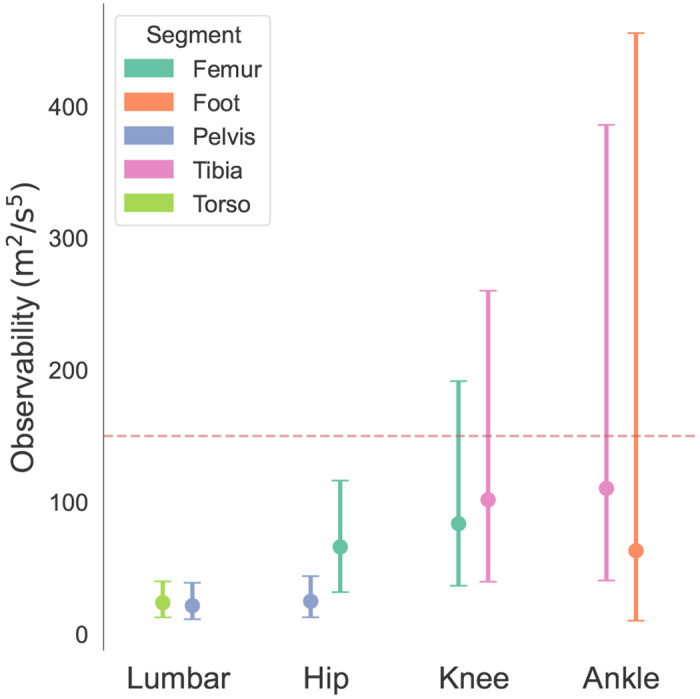
Median and inter-quartile range of observability for each joint, calculated from each segment. The applied threshold value of 150 is visualized as a red line.

The distribution of underlying sensor readings, and observability, of the evaluation dataset depended on sensor placement. The mean and standard deviation of magnetic field both increased for more distal sensors, ranging from 0.90 ± 0.10 μ*T* at the pelvis to 1.21 ± 0.74 μ*T*at the foot (see Figure 4). Similarly, the mean and standard deviation of acceleration also increased for more distal sensors, ranging from $9.94\pm 1.59\frac{m}{s^{2}}$ at the torso to $12.37\pm 5.84\frac{m}{s^{2}}$ at the foot (see Figure 4). The observability at the joints showed a similar distribution, with the lumbar joint showing the smallest median observability and inter-quartile range and the ankle showing the largest (see Figure 5). Median observability estimates for all segments fell 2 below the $150\frac{m^{2}}{s^{5}}$ threshold; however, the knee and ankle exhibited 75th percentile values that exceeded this cutoff.

## IV. Discussion

Our proposed MAJIC filter produced more accurate and consistent joint-angle estimates than competing IMU-based methods on diverse ambulation tasks, as indicated by lower median RMSE and smaller inter-quartile ranges [18], [29], [21], [20]. These improvements stem from addressing the distortion error present in global methods, and drift error present in magnetometer-free relative methods. Furthermore, our publicly available code allows researchers to easily implement the proposed method in a variety of applications.

The MAJIC filter had consistently smaller RMSE than global methods—including the reported extended Kalman filter and two complementary filters—by adapting core assumptions regarding magnetic fields and gravity (see Supplementary Figure 6). This difference in accuracy was most pronounced at the ankle joint. Large variance in the foot’s magnetic field and acceleration readings violates standard assumptions of a constant magnetic field and gravitational acceleration, leading to significant errors in global methods. Instead of referencing a static global field, the MAJIC filter compares the foot’s magnetic field to that of the tibia, which is subject to similar environmental distortions. Similarly, rather than assuming a static gravity vector, the filter enforces dynamic consistency of accelerations at the joint center to account for linear acceleration variance. By shifting from global to local references, the MAJIC filter mitigates assumption violations and halves the error observed in global methods at the ankle.

The distribution of the underlying sensor data also explains the differences in accuracy between the MAJIC filter and the magnetometer-free relative method. At the hip and lumbar joints, the magnetometer-free method showed large RMSE despite a high correlation coefficient, indicating a slowly increasing drift error. This drift stems from the low variance in pelvis and torso acceleration (see Figure 4). With small variance in acceleration, the joint observability—the metric for determining whether a relative orientation can be constructed from acceleration alone—is greatly reduced, indicating another sensor is necessary to disambiguate the relative orientation and constrain drift. The MAJIC filter’s adaptive inclusion of the magnetometer resulted in a higher correlation and a smaller RMSE, which support a lower drift error. At the ankle, where observability and magnetic distortion are both high, the magnetometer-free method performed best by leveraging the rich acceleration signal and entirely sidestepping error due to distortion. Both the magnetometer-on and MAJIC filter showed higher errors, though the MAJIC filter’s adaptive magnetometer usage provided a noticeable improvement over the naive magnetometer counterpart. Furthermore, while the MAJIC filter had worse performance than the magnetometer-free filter at the ankle, it performed as well or better at the other joints, and had a smaller range in RMSE across joints.

For practitioners who want to implement MAJIC filters in their devices or studies, there are several important things to keep in mind. First, the MAJIC filter is designed to balance two types of error: magnetic distortion error and drift error. This balance is crucial when the distribution of sensor data is unknown, as in long-term data collections. When the distribution is known in advance, the use of magnetometers should be tailored to the specific capture. If the joint is known to have large changes in acceleration, or if large magnetic distortions are known to be present, excluding the magnetometer may yield higher accuracy. Second, the MAJIC filter needs offset estimates from the IMUs to the joint centers to compute the acceleration at the joint center. We estimated the offsets from optical motion capture, though these offsets can be estimated from IMU sensor data alone using optimization if the motion used for calibration contains sufficient excitation of all joint axes [32]. Finally, our approach to adaptive magnetometer filtering can be used in tandem with methods that exclude the magnetometer if there is large variance over time to further limit the impact of magnetic distortion [22], [27].

There are many opportunities to continue improving and further characterizing the accuracy of the current method. One such opportunity is integrating advances identified in robotics and aerospace research to model magnetic field distortion. For example, the use of magnetometer arrays on a segment, inspired by drones and robotic platforms, could lead to better models of local magnetic field gradients and improve estimates of the magnetic field at the joint center [33], [34], [35]. Another opportunity is to build upon existing work that uses joint axes, and other biomechanical constraints, as sensors [36]. Incorporating biomechanical constraints into the MAJIC relative filter structure may improve accuracy. Finally, while a strong correlation exists between error in sensor fusion and error in inverse kinematics angle estimates, the direct relationship between improvements from this sensor fusion structure and the accuracy of joint angles from inverse kinematics with a musculoskeletal model remains to be fully characterized [14].

## V. Conclusion

The MAJIC filter offers an improvement in accuracy and consistency across a variety of ambulation tasks compared to standard extended Kalman filter and magnetometer-free approaches. By replacing assumptions regarding the global magnetic field and acceleration with more dynamically consistent assumptions about local references, this method bridges the gap between drift-prone and disturbance-prone algorithms. We anticipate that the increased fidelity in estimating joint angles, along with an open-source implementation, will enable more reliable analysis of human motion outside of the laboratory environment.

## Supplementary Material

1

## Figures and Tables

**Fig. 2.**
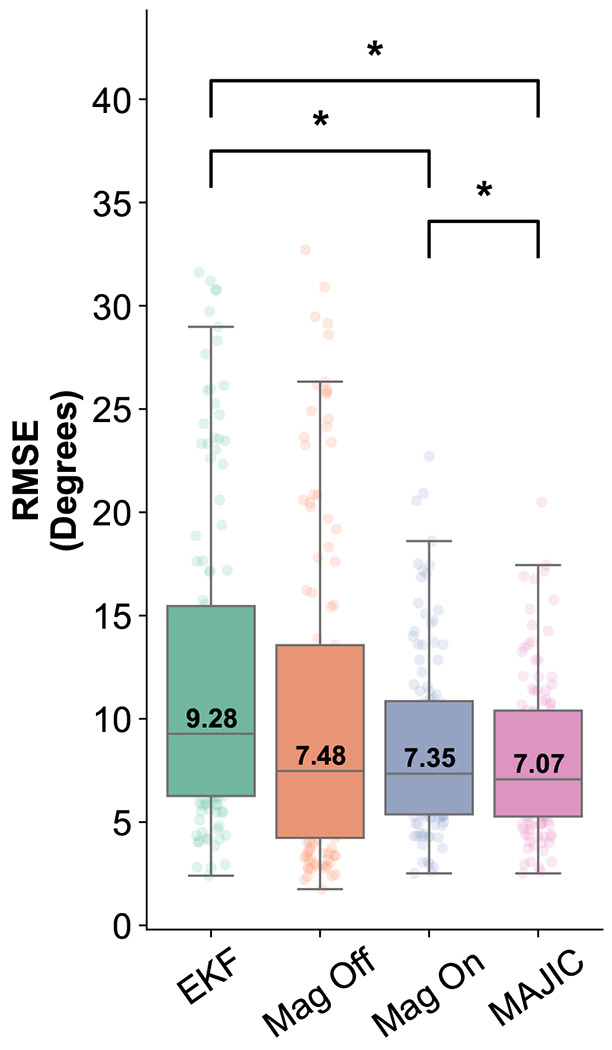
Median 3D Joint Angle RMSE Magnitude for the four tested methods: extended Kalman filter (EKF), magnetometer-free relative filter (Mag Off), magnetometer-on relative filter (Mag On) and adaptive magnetometer relative filter (MAJIC). Box plot height is equal to interquartile range with outliers defined as values exceeding 1.5 times the inter-quartile range. Statistically significant differences in RMSEs during a paired test are marked with an asterisk.

**TABLE I:** PEARSON’S CORRELATION (MEAN ± 95% CI) BY JOINT AND METHOD

Joint	EKF	Mag Off	Mag On	MAJIC
**Lumbar**	0.89 ± 0.03	0.84 ± 0.04	0.89 ± 0.02	0.89 ± 0.02
**Hip**	0.97 ± 0.01	0.94 ± 0.02	0.96 ± 0.01	0.96 ± 0.01
**Knee**	0.91 ± 0.02	0.97 ± 0.01	0.93 ± 0.02	0.93 ± 0.02
**Ankle**	0.62 ± 0.04	0.96 ± 0.01	0.72 ± 0.03	0.76 ± 0.03
