# Supplementary material for: Estimating Magnetic Field at Joint Centers Reduces Kinematic Errors in Inertial Motion Capture
Source: Res Sq. 2026 Apr 8:rs.3.rs-9337794. Preprint. [Version 1] doi: 10.21203/rs.3.rs-9337794/v1 (PMC13082144; doi:10.21203/rs.3.rs-9337794/v1)
Supplement: 1 [file NIHPPrs9337794v1-supplement-1.pdf]

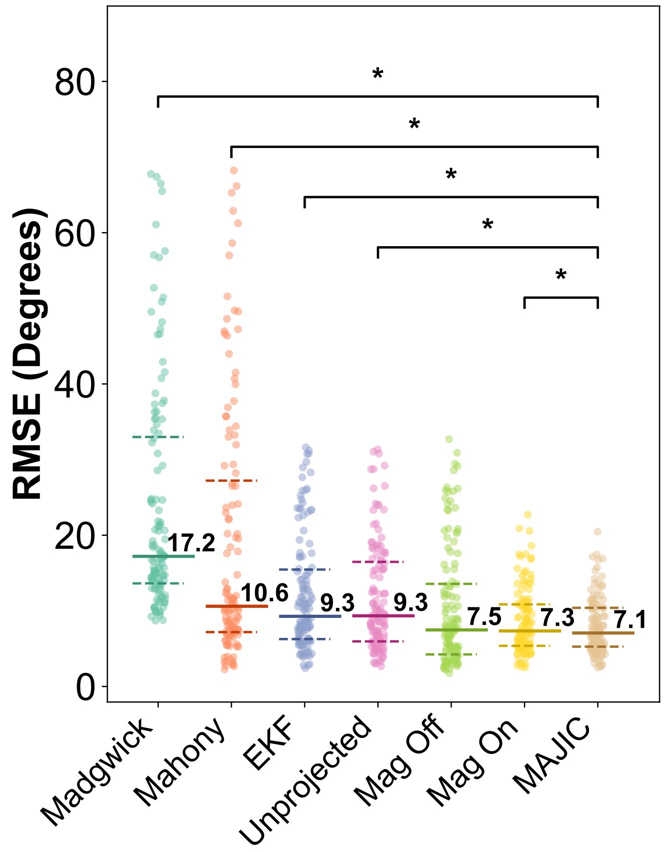

Fig. 6. Median and quartiles for additional tested methods, including 2 Complementary filters [20], [21] and the MAJIC filter without the projection of the acceleration. Statistically significant differences of each method compared to the MAJIC filter are marked with an asterisk.

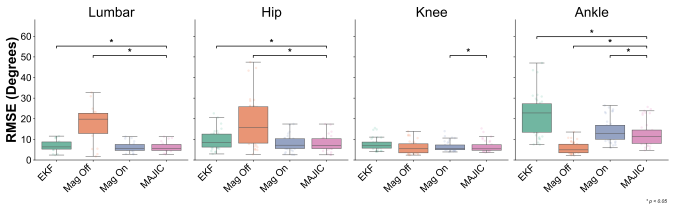

Fig. 7. Median 3D joint angle RMSE per joint for each method presented. Methods which are significantly different from the MAJIC filter are marked with an asterisk.

Appendix I
Supplementary Figures
